# Pushing the Limit of Photo-Controlled Polymerization: Hyperchromic and Bathochromic Effects

**DOI:** 10.3390/molecules29102377

**Published:** 2024-05-18

**Authors:** Zhilei Wang, Zipeng Zhang, Chenyu Wu, Zikuan Wang, Wenjian Liu

**Affiliations:** 1 Qingdao Institute for Theoretical and Computational Sciences, School of Chemistry and Chemical Engineering, Shandong University, Qingdao 266237, China; 201920282@mail.sdu.edu.cn (Z.W.); zzp13165436954@163.com (Z.Z.); 2 Max-Planck-Institut für Kohlenforschung, 45470 Mülheim an der Ruhr, Germany

**Keywords:** photo-controlled polymerization, absorption spectrum, theoretical calculations, hyperchromic effect, bathochromic effect

## Abstract

The photocatalyst (PC) zinc tetraphenylporphyrin (ZnTPP) is highly efficient for photoinduced electron/energy transfer reversible addition-fragmentation chain transfer (PET-RAFT) polymerization. However, ZnTPP suffers from poor absorbance of orange light by the so-called Q-band of the absorption spectrum (maximum absorption wavelength $\lambda_{\max}$ = 600 nm, at which molar extinction coefficient $\varepsilon_{\max}$ = $1.0\times 10^{4}$ L/(mol·cm)), hindering photo-curing applications that entail long light penetration paths. Over the past decade, there has not been any competing candidate in terms of efficiency, despite a myriad of efforts in PC design. By theoretical evaluation, here we rationally introduce a peripheral benzo moiety on each of the pyrrole rings of ZnTPP, giving zinc tetraphenyl tetrabenzoporphyrin (ZnTPTBP). This modification not only enlarges the conjugation length of the system, but also alters the $a_{1u}$ occupied π molecular orbital energy level and breaks the accidental degeneracy between the $a_{1u}$ and $a_{2u}$ orbitals, which is responsible for the low absorption intensity of the Q-band. As a consequence, not only is there a pronounced hyperchromic and bathochromic effect ($\lambda_{\max}$ = 655 nm and $\varepsilon_{\max}$ = $5.2\times 10^{4}$ L/(mol·cm)) of the Q-band, but the hyperchromic effect is achieved without increasing the intensity of the less useful, low wavelength absorption peaks of the PC. Remarkably, this strong 655 nm absorption takes advantage of deep-red (650–700 nm) light, a major component of solar light exhibiting good atmosphere penetration, exploited by the natural PC chlorophyll a as well. Compared with ZnTPP, ZnTPTBP displayed a 49% increase in PET-RAFT polymerization rate with good control, marking a significant leap in the area of photo-controlled polymerization.

## 1. Introduction

**Figure 1:**
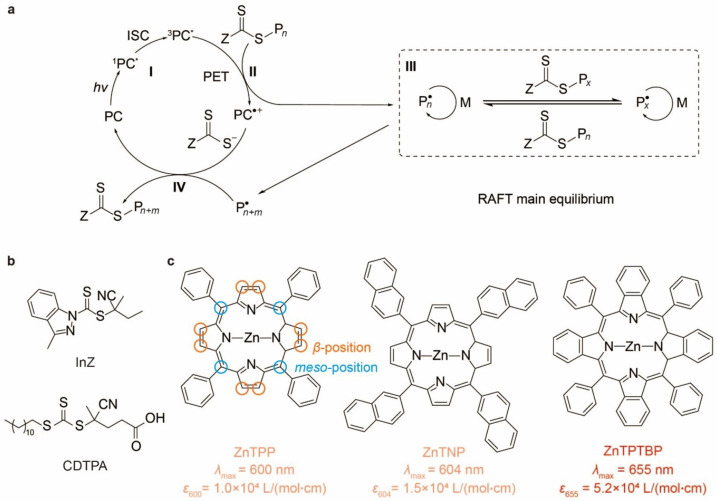
(**a**) Typical photoinduced electron/energy transfer reversible addition-fragmentation chain transfer (PET-RAFT) process. (**b**) Chemical structures of RAFT agents: 2-cyanobutan-2-yl 3-methyl-1H-indazole-1-carbodithioate (InZ) and 4-cyano-4-(dodecylsulfanylthiocarbonyl)sulfanylpentanoic acid (CDTPA). (**c**) Chemical structures of screened PCs with corresponding photophysical properties.

Sunlight has dominant contributions from the 400∼700 nm visible spectrum. Of this, deep-red (650∼700 nm) light is one of the major components of the visible sunlight, penetrating most deeply through the atmosphere. As such, chlorophyll a, the key pigment for photosynthesis, is evolved to be a deep-red-light-absorbing dye [1]. This character is sought in photo-controlled polymerization to reform the polymer industry by making use of sunlight in synthesizing well-defined polymers, but no competitive candidate has qualified. Back in 2015, chlorophyll a was initially used in photoinduced electron/energy transfer reversible addition-fragmentation chain transfer (PET-RAFT, Figure 1a) polymerization as a photocatalyst (PC) [2]. Despite its high efficiency, chlorophyll a displayed extreme photosensitivity to air and radical exposure, and degraded into colorless small molecules during polymerization [3]. In the meantime, zinc tetraphenylporphyrin (ZnTPP, Figure 1c), a chemically stable orange-light-absorbing PC, was discovered to be highly efficient for PET-RAFT polymerization, remaining the most widely used until recently [4,5,6]. Typical PET-RAFT processes (Figure 1a) for PCs, such as ZnTPP, consist of (I) photoexcitation, (II) activation, (III) chain transfer, and (IV) deactivation. In (I), the PC is excited to its singlet excited states $S_{n}$, followed by relaxing to the lowest $S_{1}$ state. Through intersystem crossing (ISC) to the triplet manifold, the lowest triplet excited state $T_{1}$ can be accessed. Owing to the spin-forbidden character of the $T_{1}$-$S_{0}$ transition, $T_{1}$ is long-lived, thereby increasing the likelihood of encountering and forming a non-covalent complex with a RAFT agent. This complexation is followed by an activation step (II), where an electron is transferred from the excited ^3^PC^*^ to the RAFT agent, leading to the cleavage of the RAFT agent’s R–S bond and the generation of a carbon-based radical (R^·^) and a dithiocarboxylate anion. R^·^ participates in the chain transfer process (III), facilitating controlled polymerization. Finally, a deactivation step (IV) occurs, resulting in the formation of a new carbon–sulfur bond between the propagating chain radical and the dithiocarboxylate, thereby completing the catalytic cycle. ZnTPP, however, suffers from a low molar extinction coefficient $\varepsilon_{\max}$ = $1.0\times 10^{4}$ L/(mol·cm) at a maximum absorption wavelength $\lambda_{\max}$ = 600 nm (longest-wavelength peak in the Q-band) from the excitation into the $S_{1}$ state, which is far worse than chlorophyll a ($\varepsilon_{\max}$ = 7 $\times \;10^{4}$∼9 $\times \;10^{4}$ L/(mol·cm) at $\lambda_{\max}$ = 660∼670 nm) [1]. It is contrasted with the strong Soret band absorption within ZnTPP, which ensures effective utilization of violet light (380∼450 nm) [7]. However, the violet light is much scattered by the atmosphere, thus somewhat depleted from the sunlight ultimately received by Earth. For its ineligibility in medium penetration, violet light is undesired in PET-RAFT polymerization [8]. Last but not least, Soret band excitation results in a higher singlet state $S_{2}$, which undergoes prompt $S_{2}$→$S_{1}$ internal conversion to yield the $S_{1}$ state [9], i.e., the same excited state as what would be obtained by exciting the Q-band. In essence, the excitation of the Soret band is no better than the Q-band for PET-RAFT polymerization, yet with significant waste of energy caused by internal conversion.

However, a strong absorbance of deep-red light has not been achieved for PET-RAFT polymerization with high efficiency, in spite of tremendous efforts [8,10,11,12]. Based on our recent advances in structure–property–performance relationships [13,14], the expansion of conjugated systems in the chromophore core enhances absorbance, leading to a hyperchromic effect. Indeed, as a macrocyclic compound, ZnTPP has a very strong $S_{0}$→$S_{2}$ absorption in the Soret band, but a weak $S_{0}$→$S_{1}$ absorption in the Q-band. By comparison, a deep-red-light-absorbing PC for PET-RAFT polymerization would require a strongly absorbing Q-band. It is therefore a non-trivial question how to selectively boost the absorption of the Q-band with a given amount of conjugated system extension, instead of solely leveraging the extended conjugated system in increasing the intensity of the Soret band. Fortunately, it is known from the early theoretical work of Gouterman [15] that the low intensity of the Q-band is due to the destructive interference between two excitations, namely, the $a_{1u}\to e_{g}$ and $a_{2u}\to e_{g}$ excitations (where we have named the orbitals based on their irreducible representations under the $D_{4h}$ group, as is conventional for the discussion of porphyrin derivatives). The two excitations have almost identical contributions to the transition dipole moment, but while in the Soret band the two excitations mix in-phase, leading to a doubled intensity, in the Q-band, they mix out-of-phase, resulting in intensity cancellation. Moreover, due to the accidental near degeneracy of the occupied $a_{1u}$ and $a_{2u}$ orbitals, the $a_{1u}\to e_{g}$ and $a_{2u}\to e_{g}$ excitations have very similar energies and thus mix with each other in close to 1:1 ratio, making the intensity of the 0–0 peak of the Q-band close to zero compared with the strong Soret band. Therefore, by attaching functional groups on the pyrrole rings to modify the $a_{1u}$ energy level, one can break the accidental degeneracy, mitigate the intensity cancellation of the Q-band by making the relative compositions of the $a_{1u}\to e_{g}$ and $a_{2u}\to e_{g}$ excitations deviate from 1:1, and enhance the Q-band absorption. As we shall see later, such a design strategy can even lead to an intensity increase in the longest wavelength vibronic peak (the 0–0 peak) of the Q-band, without also increasing the less useful 0–1 peak of the same electronic excitation (which has a slightly shorter wavelength). To further exploit the hyperchromic and bathochromic effect of enlarging conjugated systems, it is preferable to modify each of the pyrrole rings by aryl ring fusing.

Following this idea, we found that replacing the pyrrole groups of ZnTPP by benzopyrroles, resulting in zinc tetraphenyl tetrabenzoporphyrin (ZnTPTBP, Figure 1c), produces a ∼60 nm redshift of the Q-band with much enhanced absorbance (bathochromic and hyperchromic effect). As a result, ZnTPTBP has $\lambda_{\max}$ = 655 nm and $\varepsilon_{\max}$ = 5.2 × 10^4^ L/(mol·cm) in the Q-band region, showing strong deep-red-light absorbance comparable to chlorophyll a. Compared with ZnTPP, ZnTPTBP displayed a 49% increase in PET-RAFT polymerization rate with excellent control over polymerization (molecular weight dispersity $M_{w}$/$M_{n}$ = 1.12∼1.13) and satisfactory oxygen tolerance even in open-air conditions, marking a significant leap in the area of PET-RAFT polymerization. The ZnTPTBP-catalyzed PET-RAFT polymerization is then found adaptable to a plethora of solvents, as well as monomers including acrylates, acrylamides, and methacrylates.

## 2. Results and Discussion

### 2.1. Molecular Design

This manuscript aims to rationally design PCs by increasing their bathochromic and hyperchromic effects. In our recent review [13], we outlined strategies to extend the $\lambda_{\max}$ of PCs towards longer wavelengths. These strategies include heavier halogen substitution [16,17], chromophore core twisting [18,19,20], introduction of atoms with nonbonded electrons attached to the chromophore [6,21], and extension of π-conjugated systems [22,23,24]. Among these methods, extending the π-conjugated system stands out as one of the most widely used approaches for designing long-wavelength absorbing dyes. A typical strategy involves incorporating additional conjugated systems into the chromophore, which usually results in the lowest $\pi^{*}$ orbital being more stable and the highest π orbital less stable. This narrowing of the energy gap results in a bathochromic shift [13]. In addition to absorption wavelength, the intensity of absorption, indicated by a higher molar extinction coefficient ($\varepsilon_{\max}$), is also crucial, and is determined by the transition dipole moment ($\langle \Phi_{f}\left|\hat{\mu }\right|\Phi_{i}\rangle$) and the full width at half maximum of the absorption peak ($\Delta {\hat{\nu }}_{\mathrm{FWHM}}$) according to the relation $\varepsilon_{\max}$∝$\frac{f_{i\to f}}{\Delta {\bar{\nu }}_{\mathrm{FWHM}}}$, where $f_{i\to f}$=$\frac{4m_{e}\pi c}{3e^{2}\hbar \lambda }$${\left|\langle \Phi_{f}|\hat{\mu }|\Phi_{i}\rangle \right|}^{2}$ and $m_{e}$ is the electron mass, *c* is the speed of light, *e* is the charge of an electron, and *ℏ* is the Planck constant. Essentially, a higher $\langle \Phi_{f}\left|\hat{\mu }\right|\Phi_{i}\rangle$ coupled with a narrower $\Delta {\hat{\nu }}_{\mathrm{FWHM}}$ will result in a higher $\varepsilon_{\max}$ value [13].

Our choice of porphyrins as our research focus stemmed from our previous exploration of phthalocyanine derivatives [6] for PET-RAFT. In this earlier study, we observed a significant trend: as the size of the aryl rings around the tetraazaporphyrin core increased, zinc phthalocyanine exhibited a bathochromic shift of 79 nm (at $\lambda_{\max}$ = 661 nm) compared with zinc tetramethyl tetraazaporphyrin (at $\lambda_{\max}$ = 582 nm). Furthermore, when even larger aryl rings were introduced into the tetraazaporphyrin core, zinc naphthalocyanine displayed an additional 88 nm bathochromic shift (at $\lambda_{\max}$ = 749 nm) relative to zinc phthalocyanine. Simultaneously, computational analyses revealed that as the size of the aryl rings increased, both the highest occupied molecular orbital (HOMO) and the lowest unoccupied molecular orbital (LUMO) experienced elevation, albeit with the LUMO moving upward to a lesser extent. Consequently, this led to a gradual reduction in the energy gap between the HOMO and LUMO (with the HOMO to LUMO transition being the dominant component), indicating a decrease in the excitation energy required for absorptions.

Although well-controlled polymerizations with excellent livingness occur in the red to near-infrared regions using the above phthalocyanine PCs, these reactions require the presence of catalytic amounts of oxygen and an extra co-catalyst, triethylamine, through an oxygen-mediated reductive quenching pathway instead of the more common oxidative quenching pathway [6]. Due to the involvement of oxygen in the catalytic cycles, these PCs prove inert under oxygen-free conditions. Unfortunately, it is well known that oxygen can interfere with radical polymerization by intercepting the propagating radicals, leading to slow polymerization rates, long induction periods, and possibly also poor control of the molecular weight distribution. Additionally, due to their planar nature, phthalocyanine PCs demonstrate strong π-π stacking effects with extended conjugation, thus affecting their absorption properties and excited state dynamics [25]. In contrast, zinc porphyrins possess one more type of substitution sites than zinc phthalocyanines and zinc tetraazaporphyrins, since the former have carbon instead of nitrogen atoms at the meso positions, which can be easily substituted by, e.g., aryl groups (as exemplified by the phenyl and naphthyl substituents of the PCs studied in the present paper, Figure 1) to prevent π-π stacking of PC molecules through steric hindrance. Furthermore, the availability of numerous substituent positions in porphyrin derivatives may prove beneficial for achieving hyperchromic and bathochromic effects through substitution. Notably, zinc porphyrin derivatives share a core structure similar to chlorophyll a. However, chlorophyll a’s sensitivity to light and environmental exposure results in its degradation into colorless molecules during polymerization processes, limiting its widespread application, as previously discussed in the Introduction. Considering these factors, we select porphyrin derivatives as our subject, and delve into the structural–property relationships governing the photoexcitation properties, with particular focus on bathochromic and hyperchromic effects.

### 2.2. Validation of Design

Before looking into the excited state properties of the PCs, we first benchmarked our TDDFT method (PBE0-D3BJ/def2-SVP) against static–dynamic–static second-order perturbation theory (SDSPT2) [26,27,28] and experimental results (Table S1). The findings substantiated the efficacy of PBE0, as it demonstrated consistent and satisfactory accuracy across the board. Furthermore, for all three PCs studied here, we calculated the vibrationally resolved absorption spectra of the Q-band through TDDFT/PBE0-D3BJ/def2-SVP calculations on ground-state equilibrium structures using the ORCA package [29,30] (Figure S1). Our theoretical calculations successfully replicated the experimental findings regarding the relative absorption strengths of the 0–0 (long wavelength) and 0–1(short wavelength) vibronic peaks of the Q-band (Figure S1), further supporting the validity of the present computational approach.

**Figure 2:**
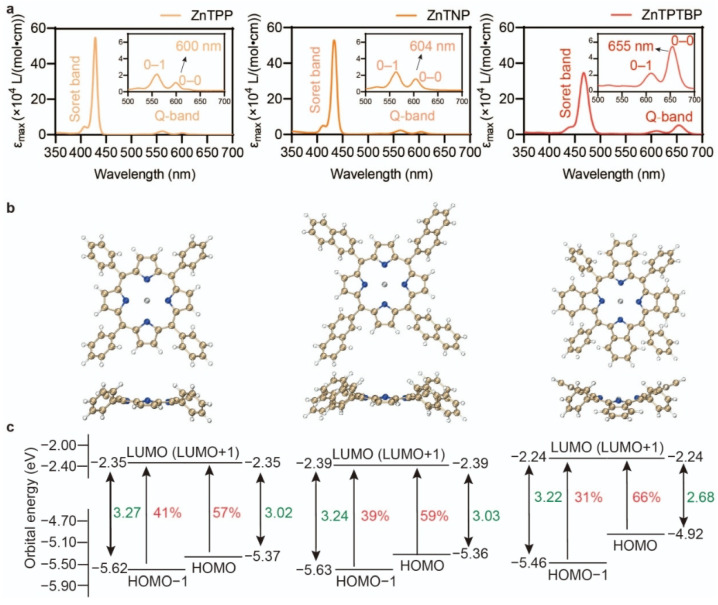

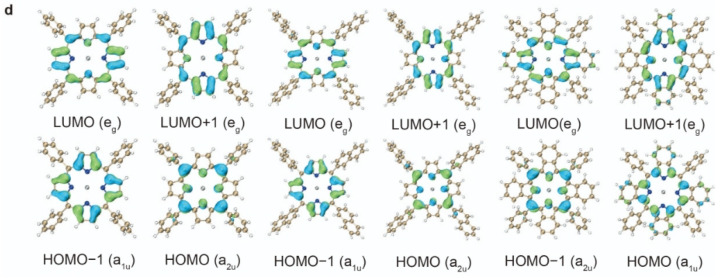
Photophysical properties of ZnTPP, ZnTNP, and ZnTPTBP. (**a**) UV–VIS absorption of PCs in DMSO with $\lambda_{\max}$ denoted; (**b**) top and side views of the molecular geometries of the PCs; (**c**) orbital energy level diagrams of HOMO-1, HOMO, LUMO, and LUMO+1 of PCs, with percentage contributions of the respective electron transitions between molecular orbitals to $S_{1}$ denoted in red; (**d**) visualization of HOMO-1, HOMO, LUMO, and LUMO+1 (isovalue = 0.05) of PCs, using VMD v1.9.4 [38] in conjunction with Multiwfn v3.8(dev) [39].

As depicted in Figure 1a, despite sharing the same chromophore, the three PCs exhibit distinct π-conjugation patterns. The ZnTPP molecule has the smallest extent of π-conjugation. Replacing the phenyl groups with naphthalene groups yields ZnTNP, where the conjugation lengths of the substituents are enlarged. Conversely, for ZnTPTBP, the extension of π-conjugation involves modifying the chromophores by substituting the pyrrole groups of ZnTPP with benzopyrrole groups. The two approaches result in very different light absorption characteristics. ZnTPP and ZnTNP exhibit similar maximum absorption wavelengths ($\lambda_{\max}$) at 600 nm and 604 nm, respectively (Table 1; Figure 2a). However, ZnTPTBP demonstrates a notable Q-band bathochromic shift with a $\lambda_{\max}$ of 655 nm, a 55 nm difference compared with ZnTPP. This observation aligns with Ruiz-Morales’s discovery that larger π-conjugation in the chromophores, as opposed to that in the substituents, is crucial for obtaining longer absorption wavelengths [24]. TDDFT results (Figure 2c) revealed that the $S_{0}$-$S_{1}$ excitations of all compounds predominantly (>95%) involve π→$\pi^{*}$ transitions between the four Gouterman orbitals, i.e., $a_{1u}$, $a_{2u}$, and the doubly degenerate $e_{g}$, as expected for typical porphyrin complexes. The bathochromic effect of ZnTPTBP relative to ZnTPP stems from the rise of the $a_{1u}$ orbital energy (by 0.70 eV), since it is the only Gouterman orbital whose direction of energy change agrees with the reduced energy gap of ZnTPTBP; the occupied $a_{2u}$ and virtual $e_{g}$ orbital energies of ZnTPTBP are lower and higher than those of ZnTPP, respectively. The increase in the $a_{1u}$ energy is strong enough to reverse the energy ordering of the $a_{1u}$ and $a_{2u}$ orbitals and, consequently, reverse the relative contributions of the $a_{1u}\to e_{g}$ and $a_{2u}\to e_{g}$ transitions in the $S_{1}$ state. From Figure 2d, the selective increase in the $a_{1u}$ orbital energy can be attributed to the fact that, among the four Gouterman orbitals, the $a_{1u}$ orbital has the greatest distribution on the pyrrole β positions, and should therefore be the most sensitive to substitutions on the pyrrole rings.

**Table 1:** Experimental and calculated photophysical properties of the PCs.

PC	Excitation	$\lambda_{\max}$ ^a^	$\varepsilon_{\max}$ ^a^	Cal. ^b^	
				$\left|\langle \Phi_{f}\left|\hat{\mu }\right|\Phi_{i}\rangle \right|$	f
		nm	$\times 10^{4}$ L/(mol·cm)	Debye	
ZnTPP	$S_{0}$-$S_{1}$ (0–0)	600	1.0	1.627	0.023
	$S_{0}$-$S_{1}$ (0–1)	560	2.1		
	$S_{0}$-$S_{2}$ (0–0)	427	55.3	10.212	1.307
	$S_{0}$-$S_{2}$ (0–1)	406	4.7		
ZnTNP	$S_{0}$-$S_{1}$(0–0)	604	1.5	2.335	0.047
	$S_{0}$-$S_{1}$ (0–1)	563	2.3		
	$S_{0}$-$S_{2}$ (0–0)	433	53.2	10.997	1.453
	$S_{0}$-$S_{2}$ (0–1)	412	5.0		
ZnTPTBP	$S_{0}$-$S_{1}$ (0–0)	655	5.2	3.623	0.105
	$S_{0}$-$S_{1}$ (0–1)	610	2.0		
	$S_{0}$-$S_{2}$ (0–0)	467	34.6	10.917	1.379
	$S_{0}$-$S_{2}$ (0–1)	442	4.3		

Note: ^a^ $\lambda_{\max}$, maximum absorption wavelength in DMSO. The concentration of PCs is 0.024 mmol·L^−1^. ^b^ The $\langle \Phi_{f}\left|\hat{\mu }\right|\Phi_{i}\rangle$ (transition dipole moment) and *f* (oscillator strength) of the PCs were calculated at the TDDFT/PBE0 [31]-D3BJ [32,33]/def2-SVP [34] level of theory, at the equilibrium structure of the ground state $S_{0}$, using the Beijing Density Functional (BDF) package [35,36,37].

In addition, the molar extinction coefficient ($\varepsilon_{\max}$) of the 0–0 Q-band absorption peak of ZnTPTBP shows a large increase (∼5 folds) compared with ZnTPP and ZnTNP. One is tempted to attribute this to the greater π-conjugation of the chromophore in ZnTPTBP: with a typical $\pi \to \pi^{*}$ excitation, the extended π-conjugation results in a broader distribution of the transition density, leading to a larger transition dipole moment ($\langle \Phi_{f}\left|\hat{\mu }\right|\Phi_{i}\rangle$) and thus increasing $\varepsilon_{\max}$. However, the frontier orbitals involved in the Q-band excitation do not significantly delocalize into the benzo groups (Figure 2d), casting doubt on this explanation. The calculation results indeed showed a 2.2-fold increase in $\langle \Phi_{f}\left|\hat{\mu }\right|\Phi_{i}\rangle$ and a 4.6-fold increase in the oscillator strength of the Q-band upon going from ZnTPP to ZnTPTBP, in reasonable agreement with the experiment. Nevertheless, the four benzo groups in ZnTPTBP have very different effects on the $\varepsilon_{\max}$ of the Soret band, decreasing (rather than increasing) it by 37%. This observation can be traced back to the breaking of the approximate degeneracy between the $a_{1u}$ and $a_{2u}$ orbitals: the gap between the $a_{1u}$ and $a_{2u}$ orbitals is doubled in ZnTPTBP (0.54 eV) compared with that in ZnTPP (0.25 eV). As a result, the difference between the contributions of the $a_{1u}\to e_{g}$ and $a_{2u}\to e_{g}$ transitions to the $S_{1}$ state is also doubled (from 16% to 35%). As the transition dipole moments of the $a_{1u}\to e_{g}$ and $a_{2u}\to e_{g}$ transitions almost cancel when their excitation vector contributions are equal [15], a two-fold increase in the contribution difference of these two transitions leads to a two-fold increase in the part of transition dipole moment that is not canceled by the linear combination of the two transitions, which suffices to explain the two-fold increase in the total transition dipole moment. We therefore believe that the increase in the $a_{1u}$–$a_{2u}$ gap, instead of the size increase in the conjugated system, is the main reason for the increase in the Q-band absorption intensity.

By contrast, the Soret band results from the in-phase linear combination of the $a_{1u}\to e_{g}$ and $a_{2u}\to e_{g}$ transitions, where the transition dipole moments of the two transitions share the same sign. Therefore, its intensity is not expected to increase due to a widening of the $a_{1u}$–$a_{2u}$ gap. The decrease in the experimental Soret band absorption intensity of ZnTPTBP compared with ZnTPP can instead be explained by peak broadening (Figure 2a), as the computed Soret band oscillator strength does not show a decrease from ZnTPP to ZnTPTBP (Table 1). Interestingly, the 0–1 peak of the Q-band has an almost constant $\varepsilon_{\max}$ across the three PCs (Table 1), unlike the 0–0 peak. This may be due to the fact that the 0–1 peak intensity of porphyrin Q-bands arises mainly due to intensity borrowing from the Soret band through the Herzberg–Teller mechanism [40], and is thus more dependent on the total absorption strength of the Soret band than that of the Q-band. Therefore, our molecular design strategy results in not only a selective intensity increase in the longest wavelength electronic transition (the Q-band), but also a selective intensity increase in the longest vibronic peak of that electronic transition (the 0–0 peak of the Q-band). Thus, the four benzo groups in ZnTPTBP were introduced in a very efficient way, in the sense that their hyperchromic effect displays exclusively in the most useful, longest wavelength absorption peak, but not in any of the shorter wavelength visible absorption peaks. This makes ZnTPTBP ideally suited for PET-RAFT applications at long wavelengths.

### 2.3. PET-RAFT Polymerization

**Figure 3:**
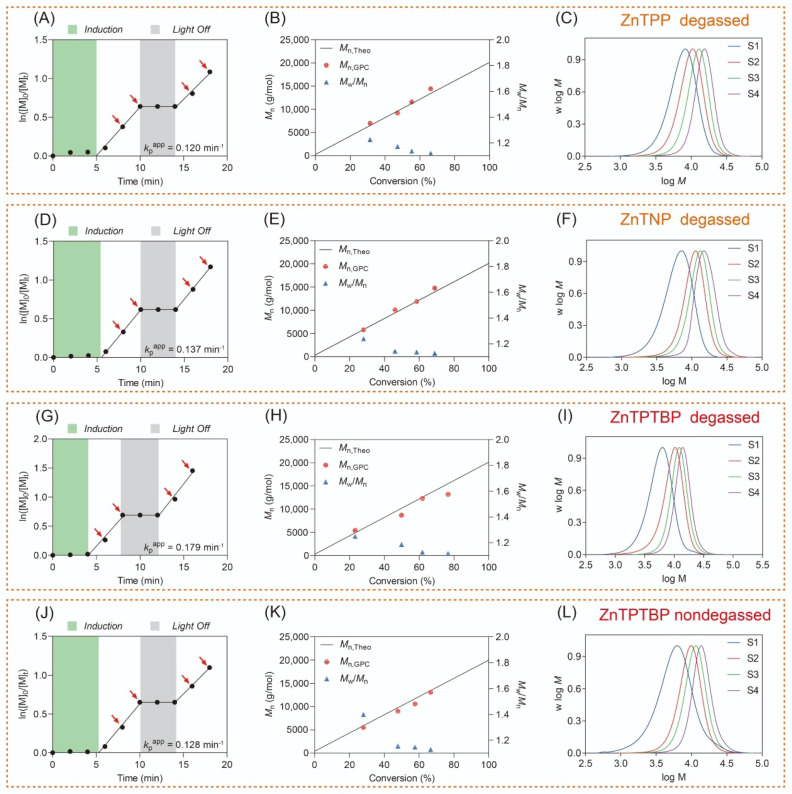
Kinetic studies for ZnTPP-, ZnTNP-, and ZnTPTBP-catalyzed PET-RAFT polymerization with InZ as the RAFT agent in the absence (**A**–**I**) and presence (**J**–**L**) of oxygen. The plot of $\mathrm{lg}M_{0}/M_{t}$ versus irradiation time (**A**,**D**,**G**,**J**), $M_{n}$ and $M_{w}$/$M_{n}$ versus monomer conversion (**B**,**E**,**H**,**K**), and evolution of normalized molecular weight distributions (MWDs) of synthesized PDMA in the process of photopolymerization (**C**,**F**,**I**,**L**).

The performance of the PCs in PET-RAFT polymerization displayed significant variability, as evidenced by the data presented in Table 2. This variability can be attributed to differences in the photophysical properties of their excited states, resulting in variations in the efficiency of the PET process. Among the three PCs, ZnTPTBP shows the fastest apparent polymerization rate ($k_{p}^{\mathrm{app}}$ = 0.179 min^−1^) compared with ZnTPP ($k_{p}^{\mathrm{app}}$ = 0.120 min^−1^) and ZnTNP ($k_{p}^{\mathrm{app}}$ = 0.137 min^−1^). The light source effectively controls the polymerization mediated by ZnTPTBP, halting polymerization promptly upon light irradiation cessation. In the absence of light, no side reactions occur (Figure 3G,J). Remarkably, ZnTPTBP also exhibits excellent resistance to oxygen, like the cases of ZnTPP and ZnTNP with InZ as the RAFT agent [41]. Despite the presence of oxygen leading to a decrease in the polymerization rate ($k_{p}^{\mathrm{app}}$ = 0.128 min^−1^) and a prolonged induction period, ZnTPTBP exhibits a polymerization rate that still exceeds that of ZnTPP-catalyzed PET-RAFT polymerization ($k_{p}^{\mathrm{app}}$ = 0.120 min^−1^) (Table 2). Additionally, oxygen has a minimal impact on the molecular weight dispersity $M_{w}$/$M_{n}$. While the initial $M_{w}$/$M_{n}$ may be poor in the presence of oxygen ($M_{w}$/$M_{n}$ = 1.40) (Figure 3K), as the conversion rate increases, the $M_{w}$/$M_{n}$ value gradually decreases ($M_{w}$/$M_{n}$ = 1.13). During the PET-RAFT polymerization catalyzed by ZnTPTBP, $M_{n,\mathrm{GPC}}$ (number average molecular weight determined by GPC) closely matches the theoretical value $M_{n,\mathrm{theo}}$, as shown in Figure 3H,K. Furthermore, we confirmed the end groups of the ZnTPTBP/InZ system using ^1^H NMR (Figures S2 and S3) and chain extension experiments (Figures S4–S6). The experimental findings demonstrate outstanding retention of end groups within the ZnTPTBP/InZ system.

**Figure 4:**
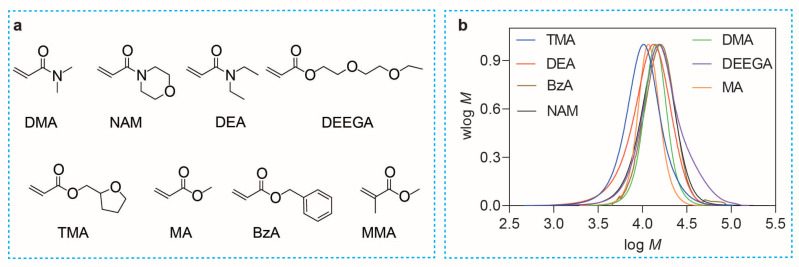
(**a**) Chemical structures of monomers. Abbreviations: N,N-dimethylacrylamide (DMA), di(ethylene glycol) ethyl ether acrylate (DEGEEA), 4-acryloylmorpholine (NAM), benzyl acrylate (BzA), methyl acrylate (MA), tetrahydrofurfuryl acrylate (TMA), N,N-diethylacrylamide (DEA), and methyl methacrylate (MMA). (**b**) Normalized MWDs of various polymers synthesized by ZnTPTBP-catalyzed PET-RAFT polymerization in Table 3.

In addition, the compatibilities of various monomers with the ZnTPTBP/InZ system were explored. The experimental findings, detailed in Table 3 and Figure 4, underscore the system’s impressive control over acrylates and acrylamides. However, our former report suggested that the synthesis of poly(methacrylate) with methyl methacrylate (MMA) as the monomer cannot be reliably controlled by the ZnTNP/InZ system, exhibiting broad molecular weight distributions (MWDs, $M_{w}$/$M_{n}$ = 1.74). This can be attributed to the difficulties of the addition of stable tertiary carbon radicals to the C=S bond, thus preventing the efficient formation of the chain transfer intermediate, due to the electron-donating nature of the indazole Z group. To test the reactivity of ZnTPTBP with MMA, another RAFT agent, CDTPA, was employed. The results demonstrate a well-controlled polymerization for MMA with ZnTPTBP as the PC (Table 3, Entry 8).

Moreover, the ZnTPTBP/InZ system achieves successful polymerization in different solvents, with dimethyl sulfoxide (DMSO) showing the fastest polymerization, reaching a conversion rate of 76.6% within 12 min (Table 4).

As a highly efficient PC, ZnTPTBP not only demonstrates an exceptionally rapid polymerization rate at a concentration of 50 ppm, but also effectively catalyzes PET-RAFT polymerization even at lower concentrations of 5 ppm and 1 ppm (Table 5), showing comparable PET-RAFT performance as chlorophyll a [2,3]. This further underlines the importance of a large $\varepsilon_{\max}$ for PET-RAFT PCs, which guarantees that a sufficient number of photons can be absorbed and turned into propagating chain radicals in a given amount of time, even if the PC concentration is extremely low.

Finally, we synthesized random copolymers with narrow molecular weight distributions via the ZnTPTBP/InZ system, as well as block copolymers with random copolymer blocks (Figures S7 and S8), which reflects the great potential of the ZnTPTBP/InZ system in preparing block and random copolymers.

## 3. Materials and Methods

### 3.1. Materials and Instruments

The monomers (DMA, DEGEEA, NAM, BzA, MA, TMA, DEA, and MMA) were purchased from Aladdin (Shanghai, China) and used after deinhibition by percolating over a column of basic alumina (Ajax Chemical, AR, Shanghai, China). The RAFT agent CDTPA (95%) was purchased from Bide Pharmatech, Shanghai, China. Zinc tetraphenylporphyrin (ZnTPP, 98%), tetranaphthylporphyrin (TNP), and tetraphenyl tetrabenzoporphyrin(TPTBP) were purchased from Bide Pharmatech (Shanghai, China). Dimethyl sulfoxide (DMSO), carbon disulfide (CS_2_), 2,2′-azobis(2-methylbutyronitrile), potassium hydroxide (KOH), zinc acetate, ethyl ether, N,N-dimethylformamide (DMF), acetonitrile (MeCN), methanol (MeOH), ethanol (EtOH), ethyl acetate (EtOAc), dichloromethane (DCM), tetrahydrofuran (THF), acetone, and petroleum ether were purchased from Sinopharm Chemical Reagent (Shanghai, China) and used as received.

Online Fourier Transform Near-Infrared (FT-NIR) spectroscopy was recorded by DONGGANG (Tianjin, China) Instrument FTIR650. It was used to monitor monomer conversions by measuring the decrease in integration of the vinylic C-H stretching overtone at ∼6200 cm^−1^. Each spectrum is composed of 16 scans with a resolution of 4 cm^−1^ (60 s per spectrum). UV–VIS spectra were recorded by a YOKE (Shanghai, China) Instrument TUV755B spectrophotometer.

Gel Permeation Chromatography (GPC): GPC with DMF as eluent was used for the molecular weight distribution characterization of polymers. The GPC modular system is composed of a Shodex (Tokyo, Japan) (KD-G 4A) 8.0 mm bead size guard column followed by a Shodex (GPC-KD-803) 6.0 mm bead size column, a differential refractive-index detector, and a UV detector. The GPC system was calibrated based on narrow molecular distributions of poly($N,N^{\prime }$-dimethylacrylamide) standards with molecular weights between 1000 and 50,000 g·mol^−1^.

Nuclear Magnetic Resonance (NMR): ^1^H NMR analysis was performed on a 400 MHz Bruker (Billerica, MA, USA) Avance NEO instrument installed with SampleXpress operating for ^1^H with chloroform-*d* (polymers and the RAFT agent) or DMSO-$d_{6}$ (the PC) as the solvent and tetramethylsilane (TMS) as the internal reference.

Mass Spectroscopy (MS): MS was performed with Thermo Scientific (Waltham, MA, USA) Q Exactive Focus, with atmospheric pressure chemical ionization (APCI) as the ion source and dichloromethane as the solvent.

Photopolymerization: Photopolymerization was carried out in 1 cm × 2 mm × 5 cm FT-NIR quartz cuvettes sealed with rubber septa. The polymerization experiments were irradiated under LEDs ($\lambda_{\max}$ = 600 and 660 nm; *I* = 10 mW·cm^−2^). Light intensity (*I*) was quantified by a DUOTONE CLOUD (Hangzhou, China) HP350C spectral power meter.

### 3.2. Synthesis of PCs (ZnTNP and ZnTPTBP) and RAFT Agent (InZ)

The synthetic methods of ZnTNP and ZnTPTBP were based on our recent work [41]. Taking ZnTPTBP as an example, 20.0 mg (24.5 μmol) of TPTBP, 6.7 mg (36.8 μmol) of anhydrous zinc acetate, and 15.0 mL of DMF were added to a glass vial with a stir bar. The reaction was carried out for 4.5 h at 75 °C. The product was monitored using UV–VIS by the complete shift of the maximum peak of absorption from reactant to product. The reaction was considered complete when the reactant absorption peak completely shifted. The resulting product was washed with deionized water 3–5 times to eliminate any excess zinc acetate. The resulting product was then dried in a vacuum drying oven at 60 °C, giving the product ZnTPTBP (14.7 mg, 16.7 μmol, 68% yield). The structure of ZnTPTBP was confirmed by ^1^H NMR spectroscopy (Figure S9).

The synthesis of InZ mainly includes two pathways (Figure S10) [41,42,43], with one of them being via bis(3-methyl-1H-indazole-1-carbothioic)disulfide. 3-Methyl-1H-indazole (2.00 g, 15.1 mmol) was added to a solution of potassium hydroxide (0.85 g, 15.1 mmol) in tetrahydrofuran (100.0 mL) at 0–5 °C. The mixture was stirred at 0–5 °C for 15 min. Thereafter, carbon disulfide (1.9 mL, 31.7 mmol) was added slowly. The reaction mixture was stirred for 5 h in ice bath. Then, I_2_ (3.83 g, 15.1 mmol) was added, and the reaction was stirred continuously for 10 h before adding sodium thiosulfate (1.20 g, 7.6 mmol) to neutralize the excess I_2_. The yellow solids were extracted with ethyl ether, which was followed by filtration. The obtained solution was washed 3 times with deionized water and then dried to receive bis(3-methyl-1H-indazole-1-carbothioic)disulfide (2.88 g, 92% yield). Then, a solution of above intermediate (2.00 g, 4.8 mmol) and 2,2′-azobis(2-methylbutyronitrile) (1.85 g, 9.6 mmol) in ethyl acetate (70.0 mL) was heated at reflux for 12 h under nitrogen atmosphere. After the removal of the volatiles in vacuo, the crude product was subjected to column chromatography with ethyl acetate (EtOAc) and petroleum ether (PE) (EtOAc:PE = 1:12, *v*/*v*) as eluent. The product 2-cyanobutan-2-yl 3-methyl-1H-indazole-1-carbodithioate was collected and dried to obtain 1.89 g yellow solid (68% yield). The structure of InZ was confirmed by MS spectroscopy (Figure S11) and ^1^H NMR spectroscopy (Figure S12).

### 3.3. General Procedure for PET-RAFT Polymerization

Taking the PET-RAFT polymerization of DMA catalyzed by ZnTPTBP under 660 nm light as an example, a stock solution of ZnTPTBP was first prepared in DMSO at a concentration of 1 mg·mL^−1^. A reaction solution consisting of DMSO (114.8 μL), *N*,*N*-dimethylacrylamide (DMA, 200.0 μL, 1.9 mmol, 50 vol%), InZ (2.8 mg, 9.7 μmol), and ZnTPTBP (85.2 μL of ZnTPP in DMSO stock solution, 0.1 μmol ZnTPP, 50 ppm relative to monomer) was prepared in a quartz cuvette (1 cm × 2 mm × 3 cm) and sealed with a rubber septum. The cuvette was wrapped with aluminum foil and deoxygenated by sparging with nitrogen for 3 min. Thereafter, the cuvette was placed under red light (660 nm) at room temperature. The monomer conversion was monitored by online FT-NIR by calculating the decrement of the vinylic C-H stretching overtone of the monomer (6100–6200 cm^−1^) at designated time points. After reaching a relatively high monomer conversion, the cuvette was removed from the light source, and aliquots were taken for GPC.

After polymerization, 0.4 mL of the poly(N,N-dimethylacrylamide) (PDMA) solution was collected and dried under reduced pressure until dryness. Subsequently, the crude solid was dissolved with minimal dichloromethane (DCM), and the resultant solution was added dropwise to 10 mL diethyl ether/petroleum ether 7:3 (*v*/*v*) for precipitation. After centrifuging, the precipitates were collected and left to dry before dissolving in minimal DCM. By repeating centrifugation a couple of times until the precipitate became a uniform and well-defined layer, the obtained solid was dried overnight under reduced pressure. An amount of 152.2 mg of purified polymer solid was obtained (78% yield).

## 4. Conclusions

By strategically introducing peripheral benzo moieties around the zinc porphyrin core, we synthesized a new PET-RAFT catalyst, ZnTPTBP, which achieved a significant increase in hyperchromic and bathochromic effects ($\lambda_{\max}$ = 655 nm and $\varepsilon_{\max}$ = $5.2\times 10^{4}$ L/(mol·cm)). This modification effectively alters the energy level of the $a_{1u}$ molecular orbital and breaks the accidental near degeneracy between the $a_{1u}$ and $a_{2u}$ orbitals, which is responsible for the low absorption intensity of the Q-band of traditional porphyrin-based PCs. Additionally, when compared with the previously studied ZnTPP, ZnTPTBP exhibited a remarkable 49% increase in the rate of PET-RAFT polymerization, representing a significant advancement in photo-controlled polymerization techniques. This innovative approach offers a viable pathway for the rational design of PCs with hyperchromic and bathochromic effects and, in particular, the selective intensity tuning of a given absorption peak of a PC for improved light absorption.

## Figures and Tables

**Table 2:** PET-RAFT polymerizations with different PCs and InZ as the RAFT agent.

Entry	PC	$k_{p}^{\mathrm{app}}$ min^−1^	$t_{\mathrm{ind}}$ min	α %	$M_{n,\mathrm{theo}}$ ^d^ g·mol^−1^	$M_{n,\mathrm{GPC}}$ g·mol^−1^	$M_{w}$/$M_{n}$	$t_{\mathrm{tot}}$ min
1	ZnTPP ^a^	0.120	4.96	66.2	13,400	14,400	1.12	12
2	ZnTNP ^a^	0.137	5.51	68.9	14,000	14,800	1.13	12
3	ZnTPTBP ^b^	0.179	4.20	76.6	15,500	13,200	1.12	12
4	ZnTPTBP ^c^	0.128	5.30	66.7	13,500	13,100	1.13	14

Note: ^a^ PET-RAFT polymerization was conducted in DMSO with DMA as the monomer under 10 mW/cm^2^ 600 nm light in the absence of oxygen. All reactions were performed at room temperature. [monomer]:[DMSO] = 1:1 *v*/*v*. A fixed reaction stoichiometry of [monomer]:[RAFT agent]:[PC] = 200:1:0.01 was used. ^b^ 10 mW/cm^2^ 660 nm in the absence of oxygen. ^c^ 10 mW/cm^2^ 660 nm in the presence of oxygen. ^d^ Theoretical molecular weight was calculated using the following equation: $M_{n,\mathrm{theo}}$ = [monomer]_0_/[RAFT]_0_ × $M_{\mathrm{monomer}}$ × α + $M_{\mathrm{RAFT}}$ agent, where [monomer]_0_, [RAFT]_0_, $M_{\mathrm{monomer}}$, α, and $M_{\mathrm{RAFT}}$ correspond to initial monomer concentration, initial RAFT concentration, the molar mass of monomer, monomer conversions determined by FT-NIR, and molar mass of RAFT agent, respectively.

**Table 3:** PET-RAFT polymerizations with different monomers and RAFT agents for the ZnTPTBP system ^a^.

Entry	Monomer	RAFT Agent	α %	$M_{n,\mathrm{theo}}$ g·mol^−1^	$M_{n,\mathrm{GPC}}$ g·mol^−1^	$M_{w}$/$M_{n}$	$t_{\mathrm{tot}}$ min
1	DMA	InZ	76.6	15,500	13,200	1.12	12
2	NAM	InZ	73.0	20,900	20,700	1.24	12
3	DEA	InZ	62.2	16,100	19,000	1.30	12
4	MA	InZ	65.3	11,500	11,300	1.10	48
5	TMA	InZ	63.3	20,100	18,200	1.27	36
6	BzA	InZ	55.3	18,200	22,000	1.11	12
7	DEEGA	InZ	71.3	27,100	21,100	1.36	36
8	MMA	CDTPA	37.0	7800	8100	1.37	40

Note: ^a^ PET-RAFT polymerization was conducted in DMSO with different monomers under 10 mW/cm^2^ 660 nm light in the absence of oxygen. All reactions were performed at room temperature. [monomer]:[DMSO] = 1:1 *v*/*v*. A fixed reaction stoichiometry of [monomer]:[RAFT agent]:[PC] = 200:1:0.01 was used.

**Table 4:** PET-RAFT polymerizations with different solvents for the ZnTPTBP/InZ system ^a^.

Entry	Solvent	Monomer	α %	$M_{n,\mathrm{theo}}$ g·mol^−1^	$M_{n,\mathrm{GPC}}$ g·mol^−1^	$M_{w}$/$M_{n}$	$t_{\mathrm{tot}}$ min
1	DMSO	DMA	76.6	15,500	13,200	1.12	12
2	MeOH	DMA	45.2	9300	9900	1.17	24
3	DMF	DMA	56.1	11,400	11,600	1.14	12
4	MeCN	DMA	74.9	15,100	14,700	1.12	24
5	EtOH	DMA	72.2	14,600	13,900	1.14	24
6	EtOAc	DMA	51.5	10,600	11,700	1.14	24
7	THF	DMA	57.8	11,700	12,100	1.21	24
8	DCM	DMA	64.8	13,100	12,600	1.15	24

Note: ^a^ PET-RAFT polymerization was conducted in different solvents, with DMA as the monomer for ZnTPTBP/InZ under 10 mW/cm^2^ 660 nm light in the absence of oxygen. All reactions were performed at room temperature. [monomer]:[DMSO] = 1:1 *v*/*v*. A fixed reaction stoichiometry of [monomer]:[RAFT agent]:[PC] = 200:1:0.01 was used.

**Table 5:** PET-RAFT polymerizations with different PC loadings for the ZnTPTBP/InZ system.

Entry	PC Loading ppm	$k_{p}^{\mathrm{app}}$ min^−1^	$t_{\mathrm{ind}}$ min	α %	$M_{n,\mathrm{theo}}$ ^a^ g·mol^−1^	$M_{n,\mathrm{GPC}}$ g·mol^−1^	$M_{w}$/$M_{n}$	$t_{\mathrm{tot}}$ min
1	25	0.110	4.78	70.9	14,400	14,200	1.09	16
2	10	0.071	5.11	70.4	13,700	14,200	1.14	22
3	5	0.057	6.57	67.5	13,700	15,100	1.13	28
4	1	0.019	31.14	65.3	13,200	12,800	1.08	88

Note: ^a^ PET-RAFT polymerization was conducted in DMSO with DMA as the monomer under 10 mW/cm^2^ 660 nm light in the absence of oxygen. All reactions were performed at room temperature. [monomer]:[DMSO] = 1:1 *v*/*v*. A fixed reaction stoichiometry of [monomer]:[RAFT agent] = 200:1 was used.

## Data Availability

Data are contained within the article and Supplementary Materials.

## Supplementary Materials

The following supporting information can be downloaded at https://www.mdpi.com/article/10.3390/molecules29102377/s1, Table S1: The experimental and calculated photophysical properties of the PCs.; Figure S1: Vibration resolved spectra of Q band absorption of (**a**) ZnTPP (orange line), (**b**) ZnTNP (orange line) and (**c**) ZnTPTBP (red line). The absorption spectra (grey lines) were taken in DMSO. The theoretical spectra were shifted to lower excitation energies by 0.29 eV for ZnTPP, 0.24 eV for ZnTNP and 0.21 eV for ZnTPTBP to match the experimental spectra; Figure S2: NMR spectrum of RAFT agent InZ; Figure S3: ^1^H NMR spectrum (400 MHz, CDCl_3_) of purified PDMA synthesized through ZnTPTBP-catalyzed PET-RAFT polymerization with InZ as RAFT agent in the absence of oxygen; Figure S4: ^1^H NMR spectrum (400 MHz, CDCl_3_) of purified PDMA synthesized through ZnTPTBP-catalyzed PET-RAFT polymerization with InZ as RAFT agent in the presence of oxygen; Figure S5: Molecular weight distributions of the synthesized PMA by ZnTPTBP-catalyzed PET-RAFT polymerization with InZ as RAFT agent (blue line) and PMA-*b*-PMA copolymer after the chain extension experiment (red line); Figure S6: Molecular weight distributions of the synthesized PDMA by ZnTPTBP-catalyzed PET-RAFT polymerization with InZ as RAFT agent (blue line) and PDMA-*b*-PNAM copolymer after the chain extension experiment (red line); Figure S7: Molecular weight distributions of the synthesized PDMA by ZnTPTBP-catalyzed PET-RAFT polymerization with InZ as RAFT agent (blue line) and PDMA-*b*-PMA copolymer after the chain extension experiment (red line); Figure S8: Molecular weight distributions of the synthesized PBzA-co-PNAM by ZnTPTBP-catalyzed PET-RAFT polymerization with InZ as RAFT agent (blue line) and PBzA-co-PNAM-*b*-PDEA-co-PTMA copolymer after the chain extension experiment (red line); Figure S9: Molecular weight distributions of the synthesized PBzA-co-PNAM by ZnTPTBP-catalyzed PET-RAFT polymerization with InZ as RAFT agent (blue line) and PBzA-co-PNAM-*b*-PDEA-co-PTMA copolymer after the chain extension experiment (red line); Figure S10: ^1^H NMR spectrum of ZnTPTBP; Figure S11: Synthesis of RAFT reagent InZ; Figure S12: MS spectrum of RAFT agent InZ. Refs. [44,45,46] are cited in Supplementary Materials.

## Abbreviations

The following abbreviations are used in this manuscript:

PC	photocatalyst
PET-RAFT	photoinduced electron/energy transfer reversible addition-fragmentation
	chain transfer
ZnTPP	zinc tetraphenylporphyrin
ZnTNP	zinc tetranaphenylporphyrin
ZnTPTBP	zinc tetraphenyl tetrabenzoporphyrin
HOMO	highest occupied molecular orbital
LUMO	lowest unoccupied molecular orbital
$k_{p}^{\mathrm{app}}$	apparent propagation rate
$\lambda_{\max}$	maximum absorption wavelength
$\varepsilon_{\max}$	molar extinction coefficient
$\langle \Phi_{f}\left|\hat{\mu }\right|\Phi_{i}\rangle$	transition dipole moment
*f*	oscillator strength
$M_{w}$/$M_{n}$	molecular weight dispersity
MWD	molecular weight distribution
$\Delta {\hat{\nu }}_{\mathrm{FWHM}}$	full width at half maximum of the absorption peak
