# Supplementary material for: Pushing the Limit of Photo-Controlled Polymerization: Hyperchromic and Bathochromic Effects
Source: Molecules. 2024 May 18;29(10):2377. doi: 10.3390/molecules29102377 (PMC11124407; doi:10.3390/molecules29102377)
Supplement: Supplementary file 1 [file molecules-29-02377-s001.zip › molecules-2988317-supplementary.pdf]

Supporting Information
Pushing the Limit of Photo-Controlled Polymerisation: Hyperchromic and Bathochromic Effects

Zhilei Wang,[†] Zipeng Zhang,[†] Chenyu Wu,[*,†] Zikuan Wang,[*,‡] and Wenjian Liu[*,†]

†*Institute of Frontier Chemistry, School of Chemistry and Chemical Engineering, Shandong University, Qingdao, China*

‡*Max-Planck-Institut für Kohlenforschung, Mülheim an der Ruhr, Germany*

E-mail: w@sdu.edu.cn(C.W.); zwang@kofo.mpg.de(Z.W.); liuwj@sdu.edu.cn(W.L.)

Theoretical Calculation Details

Theoretical Calculation Methods

Theoretical calculations were performed using the Beijing Density Functional (BDF) package.[1–3] The geometry optimisations were conducted using the PBE0 functional[4] and def2-SVP basis set,[5] coupled with the D3BJ dispersion correction[6,7] in the gas phase. The vertical excitations of PC* at the equilibrium structure of the ground state S_0 were calculated by using TDDFT/PBE0-D3BJ/def2-SVP. The TDDFT calculation results were validated by benchmarking the results against static-dynamic-static second order perturbation theory (SDSPT2)[8–10] and experiment results. The vibrational resolved spectroscopy for Q band was conducted using TDDFT/PBE0-D3BJ/def2-SVP at the S_0 structures using the

ORCA package.[11,12] The orbital plots were obtained from wavefunctions calculated at the PBE0/def2-SVP level, and were visualized using VMD v1.9.4[13] in conjunction with Multiwfn v3.8(dev).[14]

Theoretical Analyses of Photoexcitation Properties

Before delving into theoretical analysis of photoexcitation properties, it is advisable to assess the accuracy of the calculation method. In this manuscript, we utilized the single reference DFT method and the multireference method SDSPT2, the latter of which was developed by our group and can serve as a benchmark for TD-DFT calculations.[10] Comparison of the calculation results with experimental data shows that both the PBE0 and SDSPT2 methods demonstrate good accuracy for the excitation energy of S_1, consistent with our recent research on the photophysical processes of copper(II) porphyrin. We found that the hybrid functional PBE0 provides satisfactory and consistent accuracy, aligning with our previous findings.[15] The solvent effect in the photoexcitation property calculations was described by SMD,[16,17] with DMSO as solvent.

Table S1: The experimental and calculated photophysical properties of the PCs.

PC	Excitation	λ_{max}[a] nm	ε_{max}[a] $\times 10^4$ L/(mol·cm)	Cal. λ_{max}[b] nm	Cal. λ_{max}[c] nm	Cal. component[c] %	Cal. $\langle \Phi_f \| \hat{\mu} \| \Phi_i \rangle$[c] debye	Cal. f[c]	Cal.[d] λ_{max} nm	Cal.[e] λ_{max} nm
ZnTPP	S_0-S_1 (0-0)	600	1.0	537	533	$a_{1u} \rightarrow e_g$ 41	1.627	0.023	540	559
	S_0-S_1 (0-1)	560	2.1	495		$a_{2u} \rightarrow e_g$ 57				
ZnTNP	S_0-S_1 (0-0)	604	1.5	546	541	$a_{1u} \rightarrow e_g$ 39	2.335	0.047	548	569
	S_0-S_1 (0-1)	563	2.3	507		$a_{2u} \rightarrow e_g$ 59				
ZnTPTBP	S_0-S_1 (0-0)	655	5.2	590	586	$a_{1u} \rightarrow e_g$ 31	3.623	0.105	590	621
	S_0-S_1 (0-1)	610	2.0	564		$a_{2u} \rightarrow e_g$ 66				

* Note: [a]λ_{max}, maximum absorption wavelength in DMSO. The concentration of PCs is 0.024 mmol·L^{-1}. [b]Calculation method for vibrationally resolved spectroscopy: TDDFT/PBE0-D3BJ/def2-SVP at the S_0 structures. [c]Calculation method: TDDFT/PBE0-D3BJ/def2-SVP at the S_0 structures; $\langle \Phi_f | \hat{\mu} | \Phi_i \rangle$, transition dipole moment; f, oscillator strength of the PCs . [d]Calculation method: TDDFT/PBE0-D3BJ/def2-SVP/SMD(DMSO) at the S_0 structures. [e]Calculation method: SDSPT2(4,4)/def2-SV(P) with Pople correction at the S_0 structures.

To better compare with the experimental absorption spectrum, we have computed the vibration-resolved spectroscopy of the Q-bands of ZnTPP, ZnTNP and ZnTPTBP through TDDFT/PBE0-D3BJ/def2-SVP calculations on equilibrium structures of the ground state S_0, using the ORCA package.[11,12] Figure S1 shows the calculation results. The Herzberg-Teller effect was taken into account in all the calculations.

The excited state potential energy surfaces of the S_1 states of porphyrin complexes are rather anharmonic due to Jahn-Teller effects of the doubly degenerate S_1 state (for a discussion of the related porphyrin copper(II) case, see Ref. 15). Therefore, we have found that the default adiabatic Hessian (AH) method fails to reproduce the correct vibronic spectra of the molecules studied herein, since a harmonic expansion of the S_1 potential energy surface around the S_1 equilibrium geometry fails near the S_0 geometry, where the nuclear wave packet of the absorption process is mostly localized. Since a proper anharmonic treatment is too expensive considering the sizes of the molecules studied, herein the spectra were computed using the vertical gradient (VG) approximation, where the S_1 potential energy surface is harmonically expanded around the Franck-Condon point, using the excited state gradient and the ground state Hessian. Use of the ground state Hessian is mandatory since the excited state Hessian at the Franck-Condon point would have two divergent eigenvalues, due to the Jahn-Teller conical intersection. As we can see from Figure S1, apart from an underestimation of the 0-0 peak intensity of ZnTPP, our theoretical approach reproduces the splittings and the intensities of the vibrationally resolved spectra rather well.

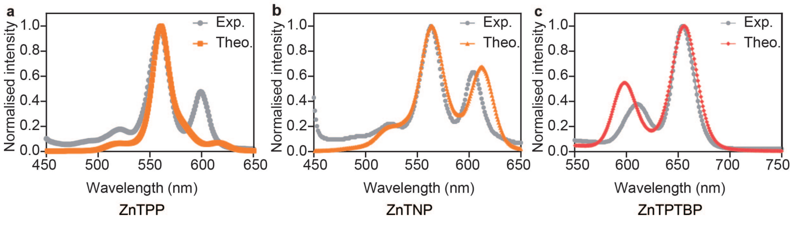

Figure S1: Vibration resolved spectra of Q band absorption of **a**) ZnTPP (orange line), **b**) ZnTNP (orange line) and **c**) ZnTPTBP (red line). The absorption spectra (grey lines) were taken in DMSO. The theoretical spectra were shifted to lower excitation energies by 0.29 eV for ZnTPP, 0.24 eV for ZnTNP and 0.21 eV for ZnTPTBP to match the experimental spectra.

Additional Results

Figure S2 shows the ^{1}H NMR spectrum (400 MHz, $CDCl_3$) of RAFT agent InZ.

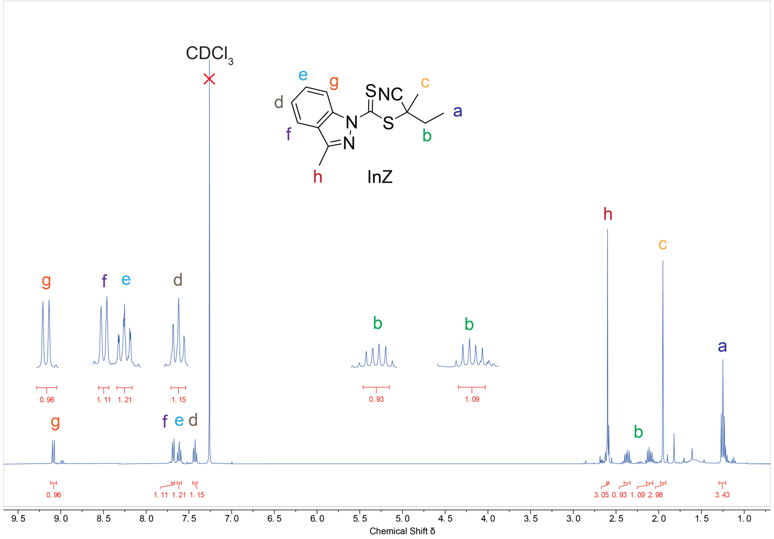

Figure S2: ^{1}H NMR spectrum of RAFT agent InZ.

Figure S3 shows the ^{1}H NMR spectrum (400 MHz, $CDCl_3$) of purified PDMA synthesized through ZnTPTBP-catalyzed PET-RAFT polymerization with InZ as RAFT agent in the absence of oxygen. The polymerization was performed at room temperature using [DMA]:[InZ]:[ZnTPTBP] = 100:1:0.01 in DMSO at [DMA]/[DMSO] = 50/50 (v/v). The solution was irradiated under 660 nm light for 18 min, and the monomer conversion was 52.0%. After polymerization, 0.4 mL of the poly(N,N'-dimethylacrylamide) (PDMA) solution was collected and dried under reduced pressure until dryness. Subsequently, the crude solid was dissolved with minimal dichloromethane, and the resultant solution was added dropwise to 8 mL diethyl ether/petroleum spirit 3:7 (v/v) for precipitation. After centrifuging, the precipitates were collected and left to dry before dissolving in minimal dichloromethane. By repeating centrifugation a couple of times until the precipitate became a uniform and well-defined layer, the obtained solid was dried overnight under reduced pressure. 83.9 mg purified polymer solid was obtained (67% yield). Approximately 10 mg of the purified polymer solid was dissolved in 0.6 mL of $CDCl_3$, and transferred to an NMR tube. The theoretical number

average molecular weight of the polymer obtained through the conversion rate is $M_{n,theo}$ = 5400 g mol^{-1}, while the number average molecular weight obtained through ^{1}H NMR is $M_{n,NMR}$ = 5300 g mol^{-1}.

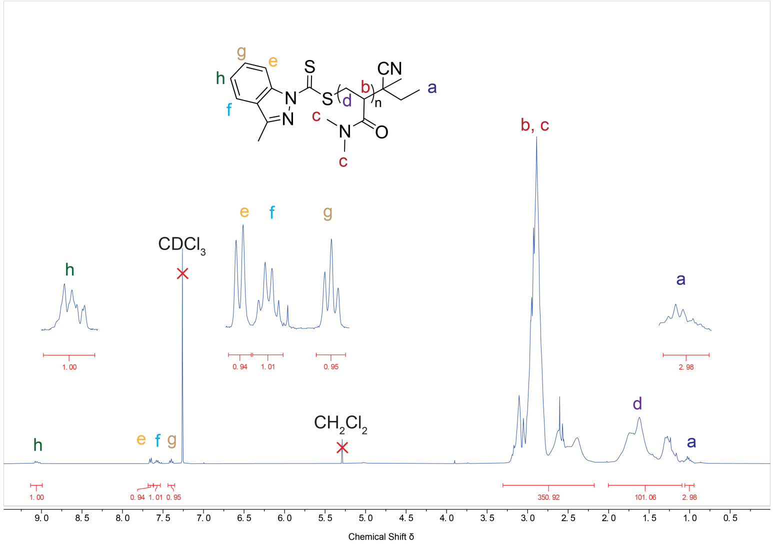

Figure S3: ^{1}H NMR spectrum (400 MHz, $CDCl_3$) of purified PDMA synthesized through ZnTPTBP-catalyzed PET-RAFT polymerization with InZ as RAFT agent in the absence of oxygen.

Figure S4 shows the ^{1}H NMR spectrum (400 MHz, $CDCl_3$) of purified PDMA synthesized through ZnTPTBP-catalyzed PET-RAFT polymerization with InZ as RAFT agent in the presence of oxygen. The polymerization was performed at room temperature using [DMA]:[InZ]:[ZnTPTBP] = 100:1:0.01 in DMSO at [DMA]/[DMSO] = 50/50 (v/v). The solution was irradiated under 660 nm light for 18 min, and the monomer conversion was 47.6%. The polymer was purified in the same way as the polymer obtained in the absence of oxygen (*vide supra*). Approximately 10 mg of the purified polymer solid was dissolved in 0.6 mL of $CDCl_3$, and transferred to an NMR tube. The theoretical number average molecular weight of the polymer obtained through the conversion rate is $M_{n,theo}$ = 5000 g mol^{-1}, while the number average molecular weight obtained through ^{1}H NMR is $M_{n,NMR}$ = 4400 g mol^{-1}.

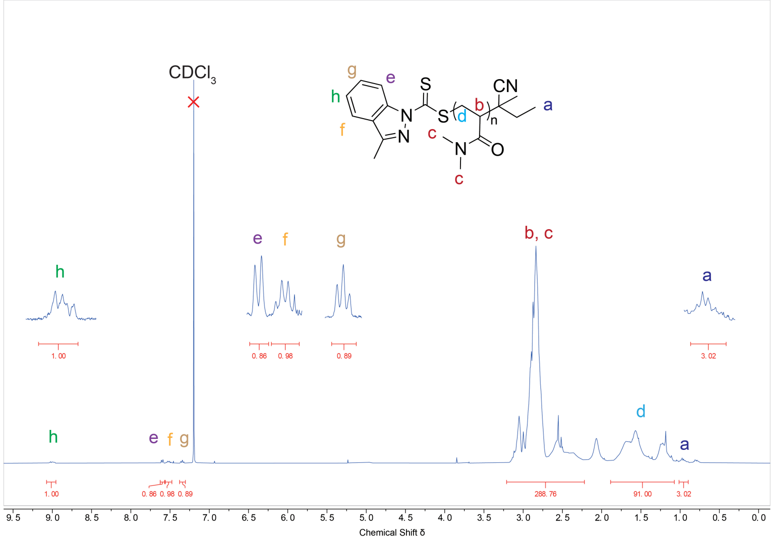

Figure S4: ^{1}H NMR spectrum (400 MHz, $CDCl_3$) of purified PDMA synthesized through ZnTPTBP-catalyzed PET-RAFT polymerization with InZ as RAFT agent in the presence of oxygen.

Figure S5 shows the molecular weight distributions of the synthesized PMA by ZnTPTBP-catalyzed PET-RAFT polymerization with InZ as RAFT agent (blue line) and PMA-*b*-PMA copolymer after the chain extension experiment (red line). The synthesis of the first block was performed using a fixed reaction stoichiometry of [MA]:[InZ]:[ZnTPBP] = 100:1:0.01, under 10 mW·cm^{-2} 660 nm irradiation for 25 min in the presence of oxygen (monomer conversion = 64.3%); chain extension was performed using a fixed reaction stoichiometry of [MA]:[PMA-RAFT agent]:[ZnTPTBP] = 300:1:0.01, under 10 mW·cm^{-2} 660 nm irradiation for 20 min in the presence of oxygen (monomer conversion = 59.9%).

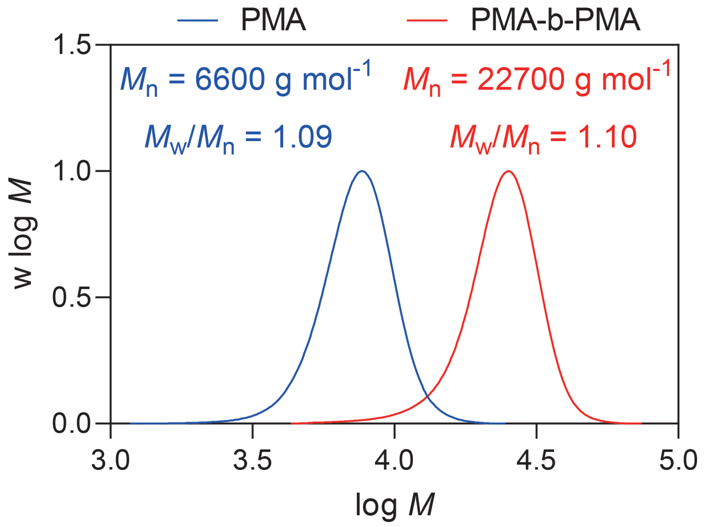

Figure S5: Molecular weight distributions of the synthesized PMA by ZnTPTBP-catalyzed PET-RAFT polymerization with InZ as RAFT agent (blue line) and PMA-*b*-PMA copolymer after the chain extension experiment (red line).

Figure S6 shows the molecular weight distributions of the synthesized PDMA by ZnTPTBP-catalyzed PET-RAFT polymerization with InZ as RAFT agent (blue line) and PDMA-*b*-PNAM copolymer after the chain extension experiment (red line). The synthesis of the first block was performed using a fixed reaction stoichiometry of [DMA]:[InZ]:[ZnTPTBP] = 100:1:0.01, under 10 mW·cm^{-2} 660 nm irradiation for 18 min in the presence of oxygen (monomer conversion = 52.0%); chain extension was performed using a fixed reaction stoichiometry of [NAM]:[PDMA-RAFT agent]:[ZnTPTBP] = 300:1:0.01, under 10 mW·cm^{-2} 660 nm irradiation for 6 min in the presence of oxygen (monomer conversion = 62.6%).

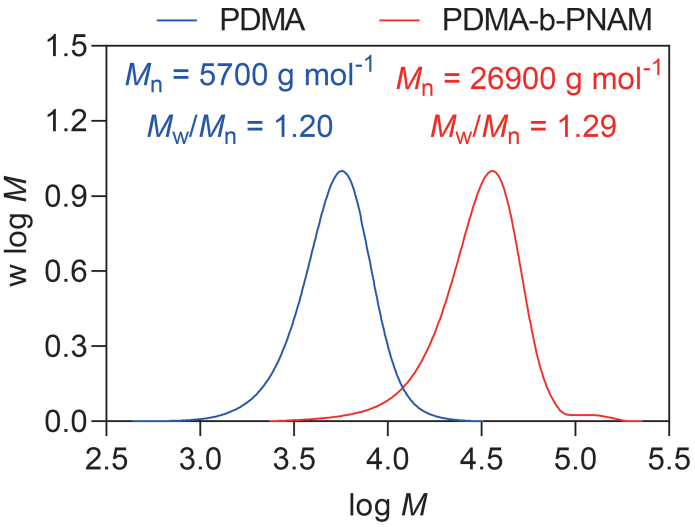

Figure S6: Molecular weight distributions of the synthesized PDMA by ZnTPTBP-catalyzed PET-RAFT polymerization with InZ as RAFT agent (blue line) and PDMA-*b*-PNAM copolymer after the chain extension experiment (red line).

Figure S7 shows the molecular weight distributions of the synthesized PDMA by ZnTPTBP-catalyzed PET-RAFT polymerization with InZ as RAFT agent (blue line) and PDMA-*b*-PMA copolymer after the chain extension experiment (red line). The synthesis of the first block was performed using a fixed reaction stoichiometry of [DMA]:[InZ]:[ZnTPTBP] = 50:1:0.01, under 10 mW·cm^{-2} 660 nm irradiation for 20 min in the presence of oxygen (monomer conversion = 65.7%); chain extension was performed using a fixed reaction stoichiometry of [MA]:[PDMA-RAFT agent]:[ZnTPTBP] = 300:1:0.01, under 10 mW·cm^{-2} 660 nm irradiation for 15 min in the presence of oxygen (monomer conversion = 69.7%).

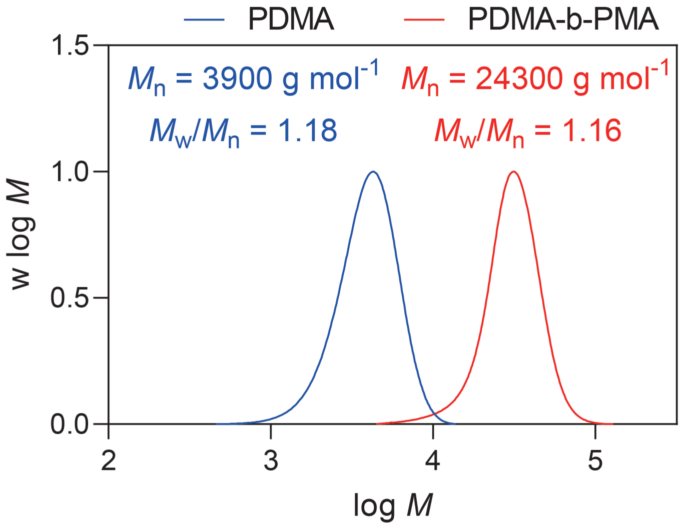

Figure S7: Molecular weight distributions of the synthesized PDMA by ZnTPTBP-catalyzed PET-RAFT polymerization with InZ as RAFT agent (blue line) and PDMA-*b*-PMA copolymer after the chain extension experiment (red line).

Figure S8 shows the molecular weight distributions of the synthesized PBzA-*co*-PNAM by ZnTPTBP-catalyzed PET-RAFT polymerization with InZ as RAFT agent (blue line) and PBzA-*co*-PNAM-*b*-PDEA-*co*-PTMA copolymer after the chain extension experiment (red line). The synthesis of the first block was performed using a fixed reaction stoichiometry of [BzA]:[NAM]:[InZ]:[ZnTPTBP] = 50:50:1:0.01, under 10 mW·cm^{-2} 660 nm irradiation for 15 min in the presence of oxygen (monomer conversion = 48.0%); chain extension was performed using a fixed reaction stoichiometry of [DEA]:[TMA]:[PBzA-*co*-PNAM-RAFT agent]:[ZnTPTBP] = 50:50:1:0.01, under 10 mW·cm^{-2} 660 nm irradiation for 15 min in the presence of oxygen (monomer conversion = 54.0%).

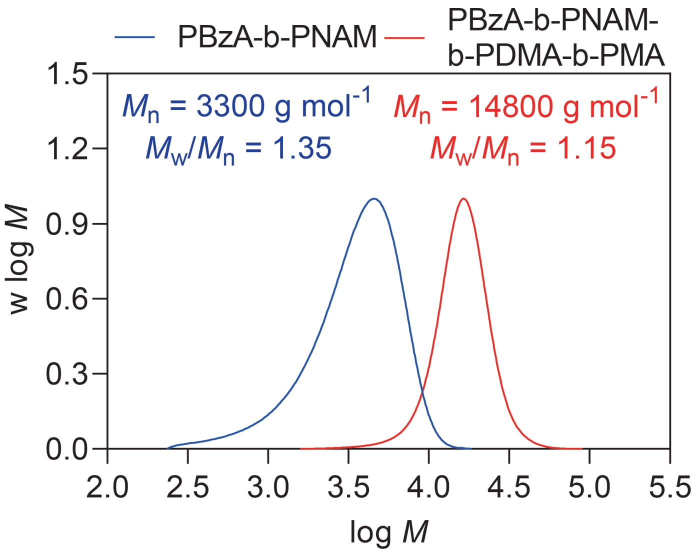

Figure S8: Molecular weight distributions of the synthesized PBzA-*co*-PNAM by ZnTPTBP-catalyzed PET-RAFT polymerization with InZ as RAFT agent (blue line) and PBzA-*co*-PNAM-*b*-PDEA-*co*-PTMA copolymer after the chain extension experiment (red line).

Figure S9 shows the molecular weight distributions of the synthesized PBzA-*co*-PNAM by ZnTPTBP-catalyzed PET-RAFT polymerization with InZ as RAFT agent (blue line) and PBzA-*co*-PNAM-*b*-PDEA-*co*-PTMA copolymer after the chain extension experiment (red line). Experimental conditions: The synthesis of the first block was performed using a fixed reaction stoichiometry of [BzA]:[NAM]:[InZ]:[ZnTPTBP] = 50:50:1:0.01, under 10 mW·cm^{-2} 660 nm irradiation for 13 min in the presence of oxygen (monomer conversion = 41.9%); chain extension was performed using a fixed reaction stoichiometry of [DMA]:[MA]:[PBzA-*co*-PNAM-RAFT agent]:[ZnTPTBP] = 50:50:1:0.01, under 10 mW·cm^{-2} 660 nm irradiation for 10 min in the presence of oxygen (monomer conversion = 64.1%).

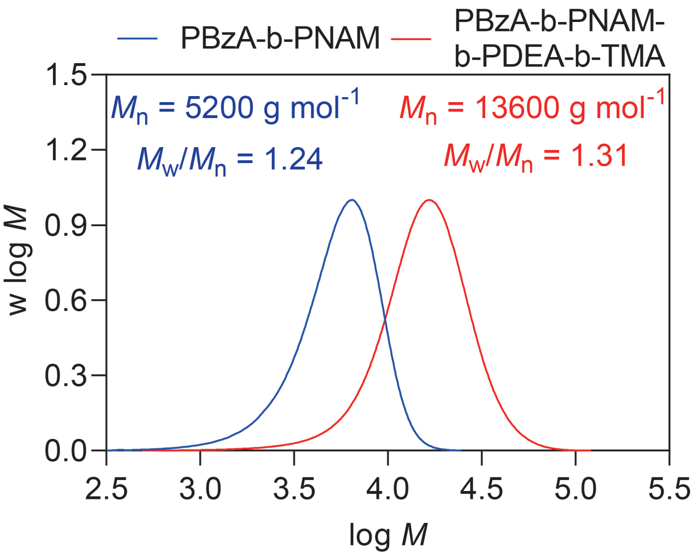

Figure S9: Molecular weight distributions of the synthesized PBzA-*co*-PNAM by ZnTPTBP-catalyzed PET-RAFT polymerization with InZ as RAFT agent (blue line) and PBzA-*co*-PNAM-*b*-PDEA-*co*-PTMA copolymer after the chain extension experiment (red line).

Figure S10 shows the ^{1}H NMR spectrum (400 MHz, DMSO-d_6) of ZnTPTBP.

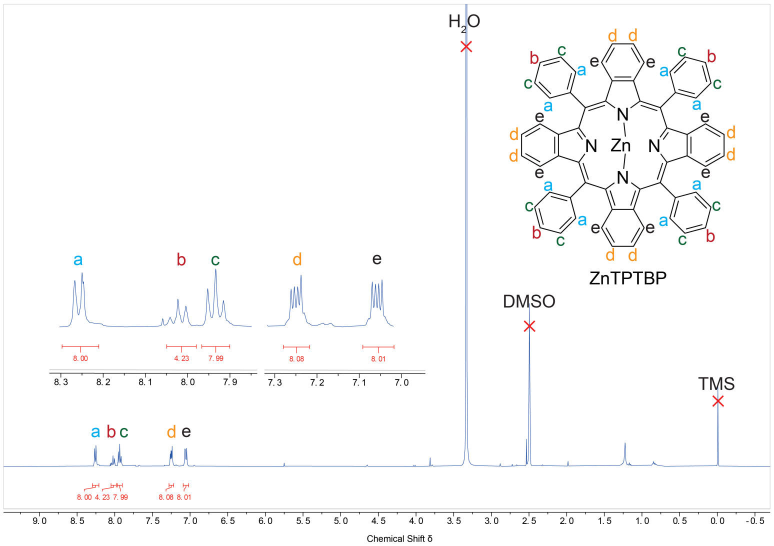

Figure S10: ^{1}H NMR spectrum of ZnTPTBP.

Figure S11 shows the two step synthesis of InZ.[18–20]

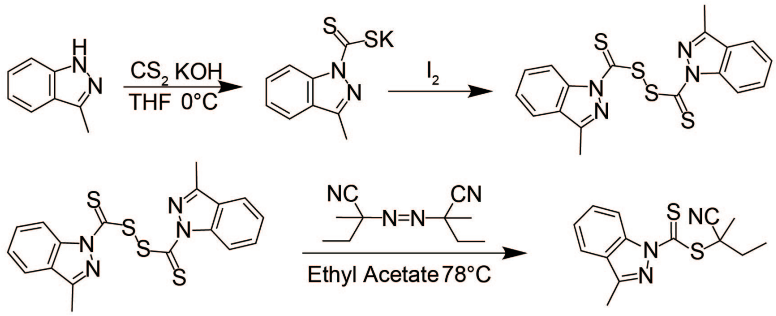

Figure S11: Synthesis of RAFT reagent InZ.

Figure S12 shows the mass spectrum (MS) of RAFT agent InZ. The MS was measured on a Thermo Scientific Q Exactive Focus spectrometer, with an APIC ion source and dichloromethane as solvent.

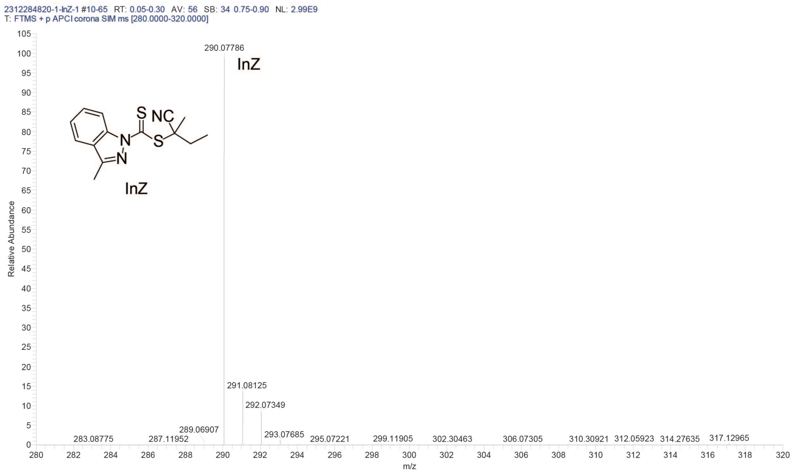

Figure S12: MS spectrum of RAFT agent InZ.

Molecular Coordinates(Å)

Molecular Coordinates of ZnTPP, ZnTNP and ZnTPTBP are shown.

ZnTPP

Ground state

C 2.51486634 3.47790242 0.13936067

C 1.24548540 2.79888751 0.02891800

N 1.44879361 1.44879361 -0.02316000

C 2.79888751 1.24548540 0.02891800

C 3.47790242 2.51486634 0.13936067

C 3.44931382 -0.00000000 -0.00000000

C 2.79888751 -1.24548540 -0.02891800

N 1.44879361 -1.44879361 0.02316000

C 1.24548540 -2.79888751 -0.02891800

C 2.51486634 -3.47790242 -0.13936067

C 3.47790242 -2.51486634 -0.13936067

C 0.00000000 3.44931382 0.00000000

C -1.24548540 2.79888751 -0.02891800

C -2.51486634 3.47790242 -0.13936067

C -3.47790242 2.51486634 -0.13936067

C -2.79888751 1.24548540 -0.02891800

N -1.44879361 1.44879361 0.02316000

C -3.44931382 0.00000000 -0.00000000

C -2.79888751 -1.24548540 0.02891800

C -3.47790242 -2.51486634 0.13936067

C -2.51486634 -3.47790242 0.13936067

C -1.24548540 -2.79888751 0.02891800

N -1.44879361 -1.44879361 -0.02316000

C -0.00000000 -3.44931382 -0.00000000

C 4.93780636 -0.00000000 0.00000000

C 5.65245239 0.52203929 -1.08623859

C 7.04555335 0.52144381 -1.08668278

C 7.74632313 -0.00000000 0.00000000

C 7.04555335 -0.52144381 1.08668278

C 5.65245239 -0.52203929 1.08623859

C 0.00000000 7.74632313 0.00000000

C 0.52144381 7.04555335 -1.08668278

C 0.52203929 5.65245239 -1.08623859

C 0.00000000 4.93780636 0.00000000

C -0.52203929 5.65245239 1.08623859

C -0.52144381 7.04555335 1.08668278

C -0.00000000 -4.93780636 -0.00000000

C 0.52203929 -5.65245239 1.08623859

C 0.52144381 -7.04555335 1.08668278

C -0.00000000 -7.74632313 -0.00000000

C -0.52144381 -7.04555335 -1.08668278

C -0.52203929 -5.65245239 -1.08623859

C -4.93780636 0.00000000 0.00000000

C -5.65245239 0.52203929 1.08623859

C -7.04555335 0.52144381 1.08668278

C -7.74632313 0.00000000 0.00000000

C -7.04555335 -0.52144381 -1.08668278

C -5.65245239 -0.52203929 -1.08623859

H 2.64644318 4.55362002 0.22671913

H 4.55362002 2.64644318 0.22671913

H 2.64644318 -4.55362002 -0.22671913

H 4.55362002 -2.64644318 -0.22671913

H -2.64644318 4.55362002 -0.22671913

H -4.55362002 2.64644318 -0.22671913

H -4.55362002 -2.64644318 0.22671913

H -2.64644318 -4.55362002 0.22671913

H 5.10199850 0.92408905 -1.94009479

H 7.58734675 0.92716359 -1.94451419

H 8.83890623 -0.00000000 0.00000000

H 7.58734675 -0.92716359 1.94451419

H 5.10199850 -0.92408905 1.94009479

H 0.00000000 8.83890623 0.00000000

H 0.92716359 7.58734675 -1.94451419

H 0.92408905 5.10199850 -1.94009479

H -0.92408905 5.10199850 1.94009479

H -0.92716359 7.58734675 1.94451419

H 0.92408905 -5.10199850 1.94009479

H 0.92716359 -7.58734675 1.94451419

H -0.00000000 -8.83890623 -0.00000000

H -0.92716359 -7.58734675 -1.94451419

H -0.92408905 -5.10199850 -1.94009479

H -5.10199850 0.92408905 1.94009479

H -7.58734675 0.92716359 1.94451419

H -8.83890623 0.00000000 0.00000000

H -7.58734675 -0.92716359 -1.94451419

H -5.10199850 -0.92408905 -1.94009479

Zn 0.00000000 -0.00000000 0.00000000

ZnTNP Ground state

C -2.88592700 1.03073924 0.03179819

N -1.55502372 1.33464883 -0.02561425

C -3.43965181 -0.26089257 -0.00334643

C -1.45301865 2.69586511 0.03323986

Zn -0.00000000 0.00000000 -0.00027303

C -2.69602342 -1.45323498 -0.03460130

C -0.26080888 3.43968015 0.00196929

N 1.33479182 1.55523373 0.02367493

N 1.55502372 -1.33464883 -0.02561425

N -1.33479182 -1.55523373 0.02367493

C 1.03091148 2.88613704 -0.03318333

C 2.69602342 1.45323498 -0.03460130

C 2.88592700 -1.03073924 0.03179819

C 1.45301865 -2.69586511 0.03323986

C -1.03091148 -2.88613704 -0.03318333

C 3.43965181 0.26089257 -0.00334643

C 0.26080888 -3.43968015 0.00196929

C -3.27761262 -2.76873366 -0.15605072

C -2.24557164 -3.65742606 -0.15560839

H -4.33972647 -2.98097442 -0.25276214

H -2.29700147 -4.73899810 -0.25203193

C 3.27761262 2.76873366 -0.15605072

C 2.24557164 3.65742606 -0.15560839

H 4.33972647 2.98097442 -0.25276214

H 2.29700147 4.73899810 -0.25203193

C -2.76847194 3.27739560 0.15532151

C -3.65715029 2.24534099 0.15502984

H -2.98071801 4.33947265 0.25243264

H -4.73867698 2.29675990 0.25196455

C 2.76847194 -3.27739560 0.15532151

C 3.65715029 -2.24534099 0.15502984

H 2.98071801 -4.33947265 0.25243264

H 4.73867698 -2.29675990 0.25196455

C -0.37759128 4.92255975 0.00116016

C 0.10433188 5.67410839 1.05477030

C -0.98746585 5.59070904 -1.09606578

C 0.00472666 7.08764647 1.06478370

H 0.56723333 5.17107835 1.90765394

C -1.09747899 6.95953903 -1.11693147

H -1.35974703 4.99458393 -1.93219662

C -0.60882386 7.74820982 -0.04383316

C 0.49386409 7.86891841 2.14388004

H -1.56119046 7.46037399 -1.97082513

C -0.70646318 9.16292955 -0.03258634

C 0.38393869 9.23952840 2.12614191

H 0.96103792 7.36051550 2.99134018

C -0.22149667 9.89296324 1.02746925

H -1.17527420 9.66466753 -0.88318718

H 0.76485280 9.82979647 2.96287353

H -0.30288372 10.98245130 1.02462742

C 0.37759128 -4.92255975 0.00116016

C -0.10433188 -5.67410839 1.05477030

C 0.98746585 -5.59070904 -1.09606578

C -0.00472666 -7.08764647 1.06478370

H -0.56723333 -5.17107835 1.90765394

C 1.09747899 -6.95953903 -1.11693147

H 1.35974703 -4.99458393 -1.93219662

C -0.49386409 -7.86891841 2.14388004

C 0.60882386 -7.74820982 -0.04383316

H 1.56119046 -7.46037399 -1.97082513

C -0.38393869 -9.23952840 2.12614191

H -0.96103792 -7.36051550 2.99134018

C 0.70646318 -9.16292955 -0.03258634

C 0.22149667 -9.89296324 1.02746925

H -0.76485280 -9.82979647 2.96287353

H 1.17527420 -9.66466753 -0.88318718

H 0.30288372 -10.98245130 1.02462742

C -4.92257772 -0.37722477 -0.00192561

C -5.67438392 0.10522923 -1.05512134

C -5.59049469 -0.98695620 1.09550123

C -7.08797798 0.00646468 -1.06442245

H -5.17150551 0.56809084 -1.90811449

C -6.95938621 -1.09625259 1.11700828

H -4.99418558 -1.35965829 1.93131309

C -7.74832078 -0.60691828 0.04442336

C -7.86954720 0.49637853 -2.14295808

H -7.46004467 -1.55984248 1.97107240

C -9.16311471 -0.70364731 0.03393689

C -9.24021773 0.38737247 -2.12446214

H -7.36131564 0.96344252 -2.99058088

C -9.89342997 -0.21792714 -1.02557674

H -9.66467603 -1.17233525 0.88470997

H -9.83071388 0.76890401 -2.96075147

H -10.98297018 -0.29858768 -1.02213515

C 4.92257772 0.37722477 -0.00192561

C 5.67438392 -0.10522923 -1.05512134

C 5.59049469 0.98695620 1.09550123

C 7.08797798 -0.00646468 -1.06442245

H 5.17150551 -0.56809084 -1.90811449

C 6.95938621 1.09625259 1.11700828

H 4.99418558 1.35965829 1.93131309

C 7.86954720 -0.49637853 -2.14295808

C 7.74832078 0.60691828 0.04442336

H 7.46004467 1.55984248 1.97107240

C 9.24021773 -0.38737247 -2.12446214

H 7.36131564 -0.96344252 -2.99058088

C 9.16311471 0.70364731 0.03393689

C 9.89342997 0.21792714 -1.02557674

H 9.83071388 -0.76890401 -2.96075147

H 9.66467603 1.17233525 0.88470997

H 10.98297018 0.29858768 -1.02213515

ZnTPTBP

Ground state

C -2.96244826 -0.60248222 0.34772583

N -2.01230976 0.35923478 0.15562738

C -2.80437593 -1.95529805 0.00212923

C -2.57171891 1.59044665 0.34411991

Zn -0.00000000 -0.00000000 -0.00094164

C -1.59045769 -2.57178703 -0.34570221

C -1.95521080 2.80442937 -0.00353068

N 0.35924503 2.01229922 -0.15759047

N 2.01230976 -0.35923478 0.15562738

N -0.35924503 -2.01229922 -0.15759047

C -0.60242680 2.96257793 -0.34915528

C 1.59045769 2.57178703 -0.34570221

C 2.57171891 -1.59044665 0.34411991

C 2.96244826 0.60248222 0.34772583

C 0.60242680 -2.96257793 -0.34915528

C 2.80437593 1.95529805 0.00212923

C 1.95521080 -2.80442937 -0.00353068

C 0.02881529 4.18353878 -0.84329414

C 1.42141536 3.93554064 -0.84078752

C -0.47182486 5.39943352 -1.33651564

C 2.31262109 4.90392183 -1.33103584

C 0.42182284 6.34784270 -1.81184554

H -1.53993428 5.60273764 -1.37221860

C 1.80279672 6.10211058 -1.80899545

H 3.38546161 4.72653816 -1.36237992

H 0.04062219 7.29315342 -2.20524352

H 2.48824611 6.85770305 -2.20005951

C -0.02881529 -4.18353878 -0.84329414

C -1.42141536 -3.93554064 -0.84078752

C 0.47182486 -5.39943352 -1.33651564

C -2.31262109 -4.90392183 -1.33103584

C -0.42182284 -6.34784270 -1.81184554

H 1.53993428 -5.60273764 -1.37221860

C -1.80279672 -6.10211058 -1.80899545

H -3.38546161 -4.72653816 -1.36237992

H -0.04062219 -7.29315342 -2.20524352

H -2.48824611 -6.85770305 -2.20005951

C -4.18316253 0.02871134 0.84255384

C -3.93519746 1.42133029 0.84000979

C -5.39867895 -0.47203719 1.33660648

C -4.90330210 2.31241711 1.33103819

C -6.34679366 0.42149562 1.81269307

H -5.60189463 -1.54015530 1.37242606

C -6.10113009 1.80248152 1.80978392

H -4.72599151 3.38523879 1.36247627

H -7.29177213 0.04017914 2.20677904

H -6.85645909 2.48787140 2.20147062

C 4.18316253 -0.02871134 0.84255384

C 3.93519746 -1.42133029 0.84000979

C 5.39867895 0.47203719 1.33660648

C 4.90330210 -2.31241711 1.33103819

C 6.34679366 -0.42149562 1.81269307

H 5.60189463 1.54015530 1.37242606

C 6.10113009 -1.80248152 1.80978392

H 4.72599151 -3.38523879 1.36247627

H 7.29177213 -0.04017914 2.20677904

H 6.85645909 -2.48787140 2.20147062

C -2.80630196 4.02490886 -0.00511022

C -3.76071367 4.22262263 -1.00915429

C -2.66363860 4.99029815 0.99779350

C -4.55570064 5.36638561 -1.01279828

H -3.87445730 3.46751615 -1.79023281

C -3.46337332 6.13074200 0.99942065

H -1.91641347 4.83765500 1.77982849

C -4.41014319 6.32226779 -0.00715086

H -5.29531568 5.51187966 -1.80374884

H -3.34579535 6.87606786 1.78964081

H -5.03568582 7.21809018 -0.00786396

C 2.80630196 -4.02490886 -0.00511022

C 2.66363860 -4.99029815 0.99779350

C 3.76071367 -4.22262263 -1.00915429

C 3.46337332 -6.13074200 0.99942065

H 1.91641347 -4.83765500 1.77982849

C 4.55570064 -5.36638561 -1.01279828

H 3.87445730 -3.46751615 -1.79023281

C 4.41014319 -6.32226779 -0.00715086

H 3.34579535 -6.87606786 1.78964081

H 5.29531568 -5.51187966 -1.80374884

H 5.03568582 -7.21809018 -0.00786396

C -4.02463862 -2.80670685 0.00441681

C -4.99103152 -2.66391086 -0.99749148

C -4.22109687 -3.76163669 1.00820956

C -6.13127687 -3.46394162 -0.99831440

H -4.83937534 -1.91629814 -1.77934001

C -5.36462849 -4.55693896 1.01263764

H -3.46518640 -3.87550693 1.78850199

C -6.32156158 -4.41118769 0.00803568

H -6.87742226 -3.34621537 -1.78771476

H -5.50912785 -5.29693606 1.80340881

H -7.21722337 -5.03695769 0.00939309

C 4.02463862 2.80670685 0.00441681

C 4.22109687 3.76163669 1.00820956

C 4.99103152 2.66391086 -0.99749148

C 5.36462849 4.55693896 1.01263764

H 3.46518640 3.87550693 1.78850199

C 6.13127687 3.46394162 -0.99831440

H 4.83937534 1.91629814 -1.77934001

C 6.32156158 4.41118769 0.00803568

H 5.50912785 5.29693606 1.80340881

H 6.87742226 3.34621537 -1.78771476

H 7.21722337 5.03695769 0.00939309

References

(1) Liu, W.; Hong, G.; Dai, D.; Li, L.; Dolg, M. The Beijing Four-Component Density Functional Program Package (BDF) and Its Application to EuO, EuS, YbO and YbS. *Theor. Chem. Acc.* **1997**, *96*, 75–83.

(2) Liu, W.; Wang, F.; Li, L. The Beijing Density Functional (BDF) Program Package: Methodologies and Applications. *J. Theor. Comput. Chem.* **2003**, *2*, 257–272.

(3) Zhang, Y. et al. BDF: A Relativistic Electronic Structure Program Package. *J. Chem. Phys.* **2020**, *152*, 064113.

(4) Adamo, C.; Barone, V. Toward Reliable Density Functional Methods without Adjustable Parameters: The PBE0 Model. *J. Chem. Phys.* **1999**, *110*, 6158–6170.

(5) Schäfer, A.; Horn, H.; Ahlrichs, R. Fully Optimized Contracted Gaussian Basis Sets for Atoms Li to Kr. *J. Chem. Phys.* **1992**, *97*, 2571–2577.

(6) Grimme, S.; Antony, J.; Ehrlich, S.; Krieg, H. A Consistent and Accurate Ab Initio Parametrisation of Density Functional Dispersion Correction (DFT-D) for the 94 Elements H-Pu. *J. Chem. Phys.* **2010**, *132*, 154104.

(7) Grimme, S.; Ehrlich, S.; Goerigk, L. Effect of the Damping Function in Dispersion Corrected Density Functional Theory. *J. Comput. Chem.* **2011**, *32*, 1456–1465.

(8) Liu, W. SDS: The Static-Dynamic-Static Framework for Strongly Correlated Electrons. *Theor. Chem. Acc.* **2014**, *133*, 1481.

(9) Lei, Y.; Liu, W.; Hoffmann, M. Further Development of SDSPT2 for Strongly Correlated Electrons. *Mol. Phys.* **2017**, *115*, 2696–2707.

(10) Song, Y.; Guo, Y.; Lei, Y.; Zhang, N.; Liu, W. The Static–Dynamic–Static Family of Methods for Strongly Correlated Electrons: Methodology and Benchmarking. *Top. Curr. Chem.* **2021**, *379*.

(11) Neese, F.; Wennmohs, F.; Becker, U.; Riplinger, C. The ORCA Quantum Chemistry Program Package. *J. Chem. Phys.* **2020**, *152*, 224108.

(12) Neese, F. Software update: The ORCA program system—Version 5.0. *WIREs Comput. Mol. Sci.* **2022**, *12*, e1606.

(13) Humphrey, W.; Dalke, A.; Schulten, K. VMD: Visual Molecular Dynamics. *J. Mol. Graph.* **1996**, *14*, 33–38.

(14) Lu, T.; Chen, F. Multiwfn: A Multifunctional Wavefunction Analyzer. *J. Comput. Chem.* **2012**, *33*, 580–592.

(15) Wang, X.; Wu, C.; Wang, Z.; Liu, W. When do Tripdoublet States Fluoresce? A Theoretical Study of Copper(II) Porphyrin. *Front. Chem.* **2023**, *11*.

(16) Marenich, A.; Cramer, C.; Truhlar, D. Universal Solvation Model Based on Solute Electron Density and on a Continuum Model of the Solvent Defined by the Bulk Dielectric Constant and Atomic Surface Tensions. *J. Phys. Chem. B* **2009**, *113*, 6378–6396.

(17) Garcia-Ratés, M.; Neese, F. Effect of the Solute Cavity on the Solvation Energy and its Derivatives within the Framework of the Gaussian Charge Scheme. *J. Comput.* **2020**, *41*, 922–939.

(18) James, G.; Ivan, M.; John, T.; Graeme, M. Dithiocarbamate RAFT agents with broad applicability–the 3,5-dimethyl-1H-pyrazole-1-carbodithioates. *Poly. Chem.* **2016**, *7*, 481–492.

(19) Thang, S.; Chong, Y.; Mayadunne, R.; Moad, G.; Rizzardo, E. A Novel Synthesis of Functional Dithioesters, Dithiocarbamates, Xanthates and Trithiocarbonates. *Tetrahedron Lett.* **1999**, *40*, 2435–2438.

(20) Zhang, Z.; Yu, Y.; Boyer, C.; Wu, Z.; Wu, C. Design and Synthesis of a New

Indazole-Decorated RAFT Agent for Highly Efficient PET-RAFT Polymerisation. *Marcromolecules* **2024**, Accepted.
